# Characteristics of clinical details and endolymphatic hydrops in unilateral and bilateral Ménière's disease in a single Asian group

**DOI:** 10.3389/fneur.2022.964217

**Published:** 2022-09-13

**Authors:** Suming Shi, Wenquan Li, Dan Wang, Tongli Ren, Wuqing Wang

**Affiliations:** ^1^ENT Institute and Otorhinolaryngology Department, Eye & ENT Hospital of Fudan University, Shanghai, China; ^2^NHC Key Laboratory of Hearing Medicine, Fudan University, Shanghai, China; ^3^Department of Otolaryngology, The Second Affiliated Hospital of Soochow University, Soochow, China

**Keywords:** bilateral Ménière's disease, unilateral Ménière's disease, clinical characteristics, endolymphatic hydrops, delayed Ménière's disease

## Abstract

**Objectives:**

To elucidate the characteristics of the clinical details and endolymphatic hydrops (EH) in bilateral Ménière's disease (BMD).

**Methods:**

A total of 545 patients with definite MD were enrolled. Demographic variables; the age of onset; disease course; inner ear function; the coexistence of related disorders such as migraine, delayed MD, drop attacks, and autoimmune diseases; familial history; and characteristics of EH were analyzed.

**Results:**

In the study population, the prevalence of BMD was 15.4%. The disease duration of BMD (84.0 ± 89.6 months) was significantly longer than that of unilateral MD (UMD, 60.1 ± 94.0 months) (*P* = 0.001). As evaluated by hearing thresholds and cervical and ocular vestibular evoked myogenic potentials, inner ear functions were more deteriorated in BMD (*P* < 0.05) than in UMD. The proportions of delayed MD and a family history of vertigo were significantly larger in BMD (*P* < 0.05). EH was observed in 100% of cases on the clinically affected side and 6.1% of cases on the unaffected side.

**Conclusion:**

A low prevalence of BMD, longer disease duration, higher frequencies of delayed MD, and family history of vertigo in patients with BMD were significant findings observed in the present study. All affected ears presented with EH, and a low percentage of unaffected sides presented with EH.

## Introduction

Ménière's disease (MD) is a complex condition of the inner ear and the most common cause of episodic vertigo combined with fluctuating hearing loss, tinnitus, and aural fullness. The precise etiology is currently unknown. Unilateral MD (UMD) accounts for the majority of MD cases, and bilateral MD (BMD) has the classic symptoms of UMD combined with disequilibrium and oscillopsia from bilateral vestibular hypofunction and communication difficulties from bilateral hearing loss (1). BMD can have a profound impact on the patient's quality of life, and treatment options are very limited. To date, the frequency and related factors of BMD remain unclear, especially concerning ethnic differences in epidemiology (2, 3).

Most cases of BMD present with both ears affected sequentially, with initial unilateral symptoms evolving toward bilateral disease (1); unfortunately, the causes are not exactly known. To date, an epidemiological association between MD and migraine has been reported, and some studies proposed the hypothesis that MD is a migraine-related phenomenon. Autoimmune pathologies are considered to be related to MD, including autoimmune arthritis (4) and thyroid diseases (5). A familial predisposition to the development of MD has also been described (6), especially for BMD (7, 8), and ethnic differences were shown in epidemiologic and genetic features (9). In addition, patients with BMD were reported to have a higher frequency of delayed MD (9). The concept of delayed MD is not different from delayed endolymphatic hydrops (DEH). Because the formation of EH was unclear, delayed MD might be more reasonable. However, whether these factors are related to BMD remains unclear, and most pertinent studies had limited data or were population-based research conducted in Western countries.

The pathological hallmark of MD is endolymphatic hydrops (EH) (10). Recently, extensive use of gadolinium (Gd) contrast-enhanced MRI has enabled the depiction of EH. Therefore, all patients included in our study underwent Gd contrast-enhanced MRI because of two reasons. First, it is worthwhile to improve the diagnostic accuracy of definite MD. Second, it is beneficial for investigating the characteristics of EH in BMD.

Therefore, we performed a prospective analysis of 545 patients with definite MD to investigate the characteristics of clinical details and EH of bilateral MD and the factors associated with bilateral MD.

## Materials and methods

### Patients

A total of 545 patients (274 men, 271 women; mean age 51.1 years, SD = 13.6 years) were included from February 2016 to September 2021. The patients enrolled in the study fulfilled the the definite MD diagnostic criteria formulated by the Classification Committee of the Bárány Society. Moreover, all patients underwent 3-T MRI. Neurotologic evaluations were performed, including an electric otoscope, audiometry, and tympanometry. Demographic variables, age of onset, disease course, inner ear functions, the coexistence of related disorders, such as migraine, delayed MD, drop attacks, systemic autoimmune diseases, and familial history of vertigo, and characteristics of EH were analyzed and compared. The enrolled patients with delayed MD had been suffering from longstanding (>2 years) unilateral severe or profound hearing loss, and cases with fluctuating hearing loss, mild or moderate hearing loss, were excluded though the time passes years before the occurrence of vertigo. The medical ethics committee of the Eye, Ear, Nose, and Throat Hospital of Fudan University approved this study, and all patients signed an intravenous Gd contrast operation consent form.

### IT or IV Gd injection and MRI acquisition

Intratympanic Gd injection (IT method) and intravenous injection (IV method) were used to visualize EH in MD. In total, 74 patients underwent the bilateral IT method (11), and 471 patients underwent IV injection for a double dose (0.4 mL/kg body weight) of Gd-HP-DO3A. For the IV method, MRI was performed 4 h after the injection, and scans were performed on a 3T MRI scanner (Verio; Siemens Healthcare, Erlangen, Germany) using a 32-channel phased array receive-only coil. The parameters applied were as follows: voxel size = 0.17 × 0.17 × 0.6 mm, scan time = 15 min and 20 s, repetition time = 6,000 ms, echo time = 181 ms, inversion time = 1,850 ms, slice thickness = 0.6 mm, field of view = 160 × 160 mm, and matrix size = 768 × 768. The 3T MR imaging was used to demonstrate EH and to exclude vestibular schwannoma or other causes of vertigo and hearing loss.

### Caloric test

The bithermal caloric test was performed in a dark room and conducted with an open-loop GN Otometrics Type 1068 air irrigator combined with an electronystagmography system (both Otometrics, Taastrup, Denmark). Patients were asked to lie in a supine position with their head and back inclined at 30° from the horizontal position. Nystagmus was bilaterally evoked and recorded after irrigation of the external auditory canal with an airflow of 8 L/min at 23°C (cool) and 49°C (warm), in the following order: cool left, cool right, warm left, and warm right. The duration of each irrigation was 60 s, and the interval between two irrigations was 5 min. The maximum slow-phase velocities elicited by cool and warm air on both sides were compared, and an abnormal result of the caloric test was noted when unilateral weakness was indicated by a value difference >25%.

### Vestibular-evoked myogenic potential test

We ensured that each patient was cooperative, could contract sternocleidomastoid muscles, and had no air-bone gaps. The VEMP test was performed on patients in a supine position in a sound-proof room with a temperature of 25°C. Using air-conducted sounds, a total of 120 auditory stimuli (short tone bursts, 500 Hz) were applied to each ear *via* calibrated insert headphones. Rise/fall time and plateau time were set at 2 ms each. A Bio-Logic Navigator PRO system (Version.7.0.0 of Biologic Auditory Evoked Potential software) was used to amplify the electromyographic signals, and electrical activity was bandpass filtered (10–1,500 Hz). To keep the electrode impedance below 5 kX, the skin was pretreated with a facial scrub. An initial intensity of 95 dB nHL was applied as the initial intensity to confirm a VEMP response and was changed afterward in decrements or increments of 5 dB nHL until VEMPs were not detectable. Response thresholds were defined as the minimum stimulus intensities of the characteristic waveform. For all patients with increased threshold, delayed latency or lack of response were regarded as “abnormal VEMP response.”

#### Cervical vestibular evoked myogenic potential test

White, blue, and red electrodes (serving as the non-inverting, inverting, and common electrodes, respectively) were placed on the lower part of the suprasternal fossa, the center of the ipsilateral sternocleidomastoid muscle (SCM), which is the same as the stimulating side, and the center of the contralateral SCM, respectively. The SCM electrodes could be switched automatically, so position adjustments of the electrodes were not necessary when the test was repeated on the opposite side. Patients were required to raise their heads for an SCM contraction when they heard a sound or saw a signal from the examiner. The elicitation of cVEMP was confirmed when the characteristic P13-N23 (positive-negative waveform, negative being upward) appeared. An absence of cVEMP was noted when the typical waveforms could not be elicited or were unrepeatable. The normal value of the cVEMP threshold was set at 75 ± 5 dB nHL.

#### Ocular vestibular evoked myogenic potential test

The blue and red electrodes (the inverting and common electrodes, respectively) were placed on the pretreated skin about 1 cm beneath the right and left eyes, respectively. When recording on the left side, the white electrode (the non-inverting electrode) was placed 2 cm below the blue one; while recording on the right side, the white electrode was placed 2 cm below the red one. There was no need to reposition the blue and red electrodes because those beneath the eyes could be switched automatically. Patients were required to gaze 30° upward from the vertical position when they heard a sound or saw a gesture from the examiner. The elicitation of oVEMP was confirmed when the characteristic N10-P15 appeared (negative-positive waveform, negative being upward). An absence of oVEMP was established when the typical waveforms could not be elicited or were unrepeatable. The normal value for the oVEMP threshold was determined as 80 ± 5 dB nHL.

### Pure tone audiometry test

Hearing thresholds before Gd intravenous injection were tested in all patients. Hearing thresholds at 250, 500, 1,000, 2,000, and 4,000 Hz were evaluated.

### Statistical analysis

Statistical analyses were performed using SPSS Statistics 17 software (IBM, Chicago, IL, USA) package. Data were presented as x ± SD. The Mann-Whitney *U* test, the independent samples *t*-test, the chi-square test, and Fisher's exact test were used for data analyses. Differences were considered to be statistically significant with a *p*-value < 0.05.

## Results

### Demographics

The group consisted of 271 women and 274 men with a first-visit age of 51.1 ± 13.6 years, an age at onset of 45.6 ± 14.3 years, and a disease course of 65.1 ± 93.6 months. A total of 461 (84.6%) out of 545 enrolled patients had UMD, and 84 (15.4%) out of 545 patients had BMD (Table 1). Of 545 patients, 71 (13.0%) had comorbid migraines, 33 (6.1%) had a family history of vertigo, 36 (6.6%) had delayed MD, 15 (2.8%) had drop attacks, and only 1 (0.2%) had a systemic autoimmune disease (Table 1).

**Table 1:** Demographic and clinical characteristics of enrolled patients (*n* = 545).

**Variables**	**Values**
Visiting age, yr, mean±SD	51.1 ± 13.6
Onset age, yr, mean±SD	45.6 ± 14.3
Disease duration, m, mean±SD	65.1 ± 93.6
**Gender**, ***n*** **(%)**
Women	271 (49.7)
Men	274 (50.3)
**Affected ear**, ***n*** **(%)**
Unilateral	461 (84.6)
Bilateral	84 (15.4)
Comorbid migraines, *n* (%)	71 (13.0)
Family history of vertigo, *n* (%)	33 (6.1)
Delayed MD, *n* (%)	36 (6.6)
Drop attack, *n* (%)	15 (2.8)
Systemic autoimmune diseases, *n* (%)	1 (0.2)

As seen in Table 2, 50.3% (232/461) of patients with UMD were women and 49.7% (229/461) were men, and 46.4% (40/84) of the patients with BMD were women and 53.6% (46/84) men, with no significant sex difference between the two groups (*P* = 0.511). Patients with BMD tended to be younger at the onset of the disease (44.2 ± 15.3 years) than those with UMD (45.9 ± 14.1 years); however, it was not significantly different (*P* = 0.322). The disease duration from the onset of the first involved ear of patients with BMD (84.0 ± 89.6 months) was significantly longer than that of the first involved ear of patients with UMD (60.1 ± 94.0 months) (*P* = 0.001).

**Table 2:** Comparison of unilateral MD versus bilateral MD.

**Variables**	**UMD (*n =* 461)**	**BMD (*n =* 84)**	***P-*Value**
Visiting age, yr, mean±SD	50.9 ± 13.4	51.9 ± 14.6	0.331
Onset age, yr, mean±SD	45.9 ± 14.1	44.2 ± 15.3	0.322
Disease duration, m, mean±SD	60.1 ± 94.0	84.0 ± 89.6	0.001^**^
**Gender**, ***n*** **(%)**
Women	232 (50.3)	39 (46.4)	0.511
Men	229 (49.7)	45 (53.6)	
Comorbid migraines, *n* (%)	59 (12.8)	12 (14.3)	0.710
Family history of vertigo, *n* (%)	22 (4.8)	11 (13.1)	0.003^**^
Delayed MD, *n* (%)	24 (5.2)	12 (14.3)	0.002^**^
Drop attack, *n* (%)	12 (2.6)	3 (3.6)	0.618
Systemic autoimmune diseases, *n* (%)	0	1 (1.2)	0.019^*^
**Canal weakness, %**
First involved ear	67.1	69.0	0.840^†^
Second involved ear	0	20.7	0.000^**^^§^
**Abnormal cVEMP response, %**
First involved ear	82.2	100.0	0.045^**^^†^
Second involved ear		84.2	0.071^§^
**Abnormal oVEMP response, %**
First involved ear	82.2	100.0	0.045^**^^†^
Second involved ear		100.0	1^§^
**Hearing threshold, dB, HL, mean±SD**
First involved ear	56.5 ± 23.1	67.5 ± 28.9	0.019^**^^†^
Second involved ear		45.8 ± 20.0	0.000^**^^§^

Hearing threshold = average of 250, 500, 1,000, 2,000, 4,000 Hz.

† A *P*-value in the comparison between the affected ear in UMD and the first involved ear in BMD.

§ A *P*-value in the comparison between the first and second involved ears in BMD. UMD, unilateral Ménière's disease; BMD, bilateral Ménière's disease.

* *P* < 0.05.

** *P* < 0.01.

### Cochlear and vestibular function

The average hearing thresholds of the first involved ear in patients with BMD (67.5 ± 28.9 dB HL) were significantly higher than those of the affected side in patients with UMD (56.5 ± 23.1 dB HL, *P* = 0.019) (Table 2). In patients with BMD, the mean hearing thresholds of the first involved ear (67.5 ± 28.9 dB HL) were significantly higher than those of the second involved ear (45.8 ± 20.0 dB HL) (*P* < 0.001) (Table 2).

Caloric testing was performed in 167 patients with UMD and 29 patients with BMD. Caloric weakness was not significantly different between the first involved ear in the BMD group (20/29, 69%) and the affected ear in the UMD group (112/167, 67.1%) (*P* = 0.084). In patients with BMD, the caloric weakness in the first involved ear was significantly more deteriorated than that in the second involved ear (6/29, 20.7%) (*P* < 0.001) (Table 2). cVEMP and oVEMP were performed in 146 patients with UMD and 19 patients with BMD. The proportion of the first involved ears with abnormal response in cVEMP recordings was significantly higher in patients with BMD (BMD, 19/19, 100%; UMD, 120/146, 82.2%) (*P* = 0.045) (Table 2). The difference was not significant between the initially involved side (19/19, 100%) and the second involved side (16/19, 84.2%) in patients with BMD (*P* = 0.071). The proportion of the first involved ears with abnormal response in oVEMP recordings was significantly higher in patients with BMD (BMD, 19/19, 100%; UMD, 120/146, 82.2%) (*P* = 0.045) (Table 2). The difference was not significant between the initially involved side (19/19, 100%) and the second involved side (19/19, 100%) in patients with BMD (*P* > 0.05). Overall, cochlear and vestibular functions were more deteriorated in the first involved ear of patients with BMD than those in the affected ear of patients with UMD.

### Comorbidities

There was no significant difference in the prevalence of comorbid migraines between the two groups (UMD, 12.8%; BMD, 14.3%) (*P* = 0.710) (Table 2). In the UMD group, 22 patients (4.8%) had family members with vertigo, whereas the BMD group had 11 patients (13.1%) who had family members with vertigo; this difference was significantly different (*P* = 0.003) (Table 2). However, family members with vertigo had not yet been diagnosed with MD. The proportion of patients with delayed MD was significantly larger in the BMD group (BMD, 14.3%; UMD, 5.2%) (*P* = 0.002) (Table 2). Twelve patients (2.6%) in the UMD group had drop attacks, and three patients (3.6%) in the BMD group had drop attacks (*P* = 0.618) (Table 2). Only one patient with BMD was diagnosed with rheumatic polymyopathy (Table 2).

### Image findings

**Figure 1:**
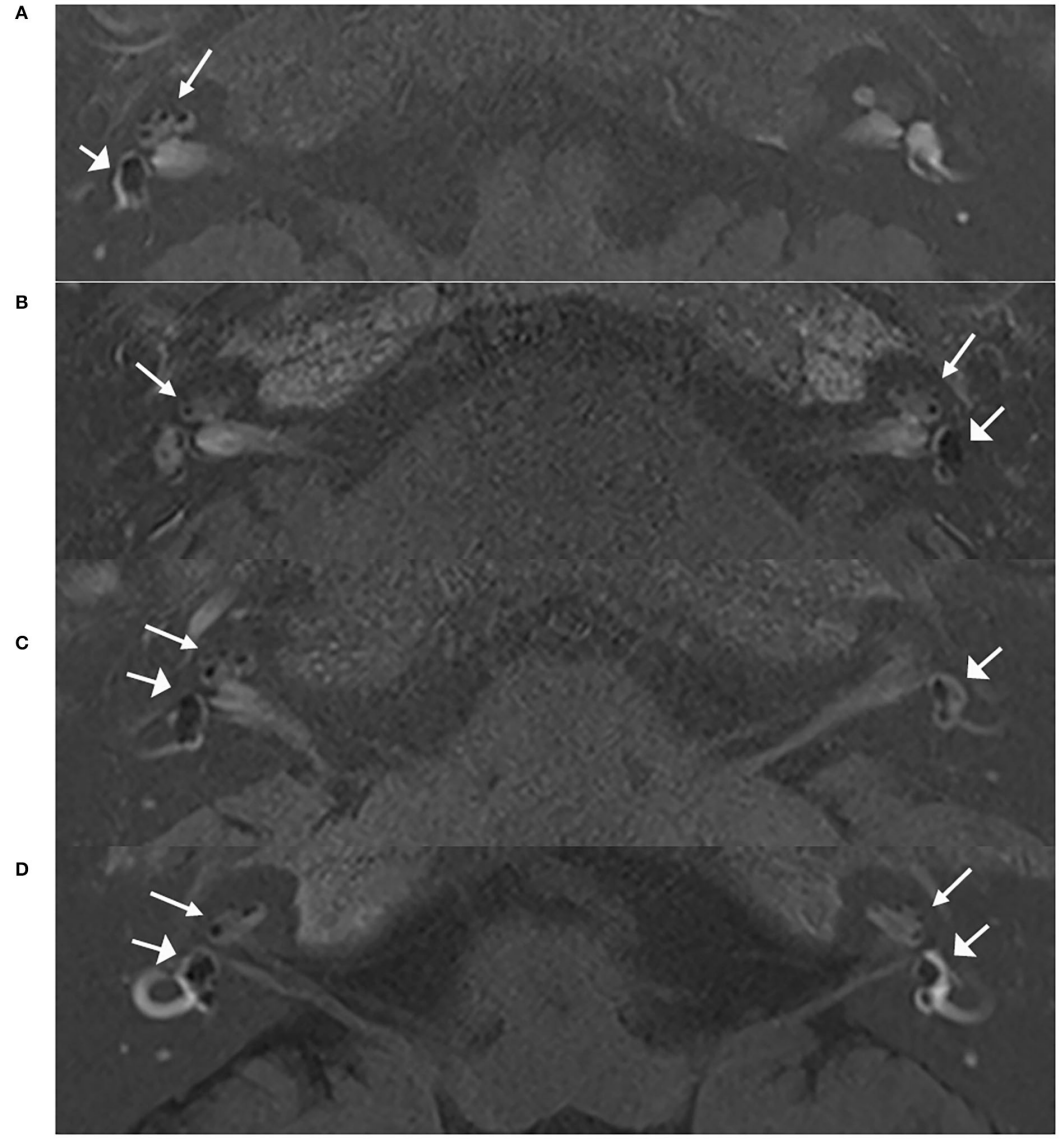
MRI scans of patients with unilateral and bilateral ELH. Images of 3D real IR performed 4 h after intravenous Gd injection. **(A)** Vestibular and cochlear ELH on the right side in a patient with unilateral MD; **(B)** Vestibular and cochlear ELH on the left side and cochlear ELH on the contralateral side in a patient with unilateral MD; **(C)** Vestibular and cochlear ELH on the right side and vestibular ELH on the contralateral side in a patient with unilateral MD; **(D)** Vestibular and cochlear ELH on the bilateral ears in a patient with bilateral MD. MD, Ménière's disease. Cochlea (thin arrows) and vestibule (broad arrows).

All patients (*n* = 545, 100%) had EH in the affected ears, including 461 cases with unilateral MD and 84 cases with bilateral MD. Among these 461 patients with UMD, 21 patients (4.6%) had EH on the contralateral side, including 11 patients with only cochlear EH, 3 patients with only vestibular EH, and 7 patients with both cochlear and vestibular EH. The extent of EH on the contralateral side was mild (Figure 1). Of the 21 cases, 9 cases had unexplained hearing loss. Notably, all ears with symptoms of hearing loss, aural fullness, or tinnitus were regarded as symptomatic. For the 84 cases with bilateral MD, all ears (168 ears) had EH, and the proportion of EH in the second involved ear of patients with BMD was significantly larger than that in the contralateral side of patients in the UMD group (*P* < 0.05) (Table 3).

**Table 3:** Percentages of endolymphatic hydrops in UMD and BMD.

**ELH, *n* (%)**	**UMD (*n =* 461)**	**BMD (*n =* 84)**	***P-*Value**
First involved ear	461 (100)	84 (100)	/
Contralateral ear	21 (4.6)	84 (100)	0.000^**^^†^

† A *P*-value in the comparison between the clinically silent ear in UMD and the second involved ear in BMD. UMD, unilateral Ménière's disease; BMD, bilateral Ménière's disease.

** *P* < 0.01.

## Discussion

We enrolled a large number of MD patients, and all patients were diagnosed by clinical criteria and MRI, ensuring the diagnostic accuracy of definite MD. The results were more persuasive compared with previous studies. Additionally, we analyzed the clinical characteristics, cochlear and vestibular functions, and features of EH of UMD and BMD, aiming to comprehensively explore the characteristics of BMD and the associated factors for the development of BMD.

The overall prevalence of BMD was 15.4%, and this value was within the range of 5.4–29% in the Asian population, as reported in previous studies (9, 12–14). The prevalence of BMD of 2–47% in the Caucasian population was higher, as reported in a previous study (15), which might indicate ethnic diversity in epidemiology (2, 3). No gender difference (man:woman = 274:271) was noted among the 545 patients, and the female preponderance in some previous studies might involve patients with vestibular migraine. Patients with BMD had a longer disease course than those with UMD; bilateral involvement occurred through metachronous progression, and the second ear was involved more than 5–10 years after the onset of first ear involvement (1, 15). The comorbid rate of migraine in MD was reported to be 22–56%, which was higher than that reported in the general population (6–17%) (16, 17). However, the prevalence rates (UMD, 12.8%; BMD, 14.3%) were lower in our study. The definite MD in the patients enrolled in our study was diagnosed by clinical features and EH, which accurately ruled out other vestibular disorders, especially easily misdiagnosed vestibular migraine. The proportion of family history of vertigo was higher in patients with BMD in our study (BMD, 13.1%; UMD, 4.8%). Genetic mechanisms have been suggested as a mechanism of detecting MD, especially for BMD. Several genes, including FAM136A, DTNA, PRKCB, SEMA3D, OTOG, and DPT (18), have been suggested but no single gene has been validated. A higher prevalence of familial MD in patients with BMD has also been reported in some studies, but other studies took a different issue (2, 9, 19). The proportion of patients with delayed MD was significantly larger in the BMD group, and the result was similar to that of a previous study (9). The underlying mechanism remains unclear, and the autoimmune pathology might involved (20, 21), predisposing ears to EH subsequently. Moreover, it also indicates that attention should be paid to delayed MD, which has the potential to become BMD. No significant difference was noted in the proportion of drop attacks between patients with BMD (3.6%) and patients with UMD (2.6%). The frequency of drop attacks varied from 3 to 19% in different studies, and it was reported to be a common phenomenon in MD, which occurred even in mild MD and complicated with syncope (22). Autoimmune pathologies were considered to be related to MD (23); however, only one patient with BMD was diagnosed with rheumatic polymyopathy, a kind of systemic autoimmune disease. Nevertheless, we cannot entirely exclude the possibility of other potential autoimmune pathologies. Cochlear and vestibular functions deteriorated more in the first involved ear of patients with BMD than in the affected side of those with UMD. The longer disease duration of patients with BMD may be the main reason. The better results of hearing thresholds and vestibular functions in the second involved ear compared with the first involved ear may further indicate the metachronous process of bilateral involvement in BMD (9). The high proportions of abnormal VEMP and the caloric tests in the second involved ear of BMD also suggest bilateral involvement (24).

All patients underwent the IT or IV method. Both methods were useful techniques for the clarification of the inner ear clinical condition through a statistical analysis of signal-intensity differences in the perilymph fluid. Of the two methods, the IV method was less invasive and ascertained the presence of EH in the bilateral labyrinth. All the 545 patients presented with EH in the affected ears. On the nonaffected side of the patients with UMD, 28 cases (6.1%) had EH, and the extent of EH in the nonaffected ears seemed to be lighter. Gu et al. (25) showed that all eight patients with bilateral definite MD had bilateral EH. Wu et al. (26) evaluated EH of both sides in 54 patients with unilateral definite MD and reported that all ears had EH on the affected sides and that nine ears had EH on the nonaffected side. Morimoto et al. reported that 48% of the cochlea and 55% of the vestibule showed EH on the nonaffected side (27). These results might indicate that EH is the hallmark of UMD and BMD, and it seems that symptoms of MD are present even after the development of EH. The percentage of EH on the nonaffected side was lower in our study (4.6%). Due to ethnic differences and different disease durations, temporary symptoms of hearing loss, aural fullness, or tinnitus might be overlooked. Moreover, it was reported that the sensitivity of MRI scans is 50% with a different technique and probably less (28), which might be a factor of the different percentage of EH. To determine whether there is a possibility of developing bilateral MD in patients with bilateral EH in unilateral MD, a longitudinal study is needed.

Our research still has some limitations. Because it was a retrospective study, not every patient was evaluated with the same vestibular tests. Therefore, more prospective studies are needed.

## Conclusion

Overall, a lower prevalence of BMD, longer disease duration, and higher frequencies of delayed MD and family history of vertigo were found in patients with BMD compared with patients with UMD. A low frequency of systemic autoimmune diseases was found in both patients with UMD and BMD. The variables of gender, comorbid migraines, and drop attacks were not significantly different between patients with BMD and UMD. All patients presented with EH on the affected ears, and a low percentage of unaffected ears presented with EH. These findings will provide information about the development of BMD. In addition, for UMD patients with those risk factors, serious considerations before aggressive treatment for the first involved ear were needed.

## Data availability statement

The original contributions presented in the study are included in the article/supplementary material, further inquiries can be directed to the corresponding author.

## Ethics statement

The studies involving human participants were reviewed and approved by the Medical Ethics Committee of the Eye, Ear, Nose, and Throat Hospital of Fudan University approved this study, and all patients signed an intravenous Gd contrast operation consent form. Written informed consent to participate in this study was provided by the participants' legal guardian/next of kin.

## Author contributions

WW provided approval for publication of the content. WW and SS agreed to be accountable for all aspects of the work in ensuring that questions related to the accuracy or integrity of any part of the work are appropriately investigated and resolved. SS drafting the work or revising it critically for important intellectual content. SS, WL, DW, and TR made substantial contributions to the conception or design of the work, the acquisition, analysis, and interpretation of data for the work. All authors contributed to the article and approved the submitted version.

## Funding

This study was supported by the National Natural Science Foundation of China (Nos. 82101222 and 81670933) and the Natural Science Foundation of Shanghai (No. 20ZR1409600).

## Conflict of interest

The authors declare that the research was conducted in the absence of any commercial or financial relationships that could be construed as a potential conflict of interest.

## Publisher's note

All claims expressed in this article are solely those of the authors and do not necessarily represent those of their affiliated organizations, or those of the publisher, the editors and the reviewers. Any product that may be evaluated in this article, or claim that may be made by its manufacturer, is not guaranteed or endorsed by the publisher.
